# Prevalence and Associated Factors Accounting for In‐School Adolescents' Serious Injuries in Bolivia: A Population‐Based Cross‐Sectional Survey

**DOI:** 10.1002/hsr2.72149

**Published:** 2026-03-23

**Authors:** Jacob Owusu Sarfo, Richmond Stephen Sorkpor, Edmond Kwesi Agormedah, Medina Srem‐Sai, Paul Obeng, Newton Isaac Gbordzoe, John Elvis Hagan

**Affiliations:** ^1^ Department of Health, Physical Education and Recreation University of Cape Coast Cape Coast Ghana; ^2^ Department of Health, Physical Education, Recreation and Sports University of Education Winneba Ghana; ^3^ Department of Business & Social Sciences Education University of Cape Coast Cape Coast Ghana; ^4^ Department of Public Health and Health Promotion Robert Gordon University Aberdeen UK; ^5^ School of Nursing and Midwifery Family Health University College Accra Ghana; ^6^ Neurocognition and Action‐Biomechanics‐Research Group, Faculty of Psychology and Sports Science Bielefeld University Bielefeld Germany

**Keywords:** adolescents, aggression and violence, Bolivia, injuries, substance use, suicidal behavior

## Abstract

**Background and Aim:**

Serious injuries are harmful actions and conduct that can contribute to serious health problems among adolescents in schools. Serious injuries account for a significant proportion of the global burden of disease in adolescents. Given the limited information on the Bolivian context, the current study aims to examine the prevalence of this phenomenon and its associated factors among school‐age adolescents in Bolivia.

**Methods:**

A cross‐sectional design was used to draw data from the 2018 Bolivia Global School‐Based Student Health Survey. This survey used a self‐administered questionnaire to collect data on adolescents' health behaviors and protective factors in Bolivia. Pearson's *χ* ^2^ and binomial logistic regression were used for the analyses.

**Results:**

The prevalence of serious injuries among 7822 Bolivian in‐school adolescents was 51.9%, with male (52%) and female (52.8%) participants experiencing serious injuries, respectively. The odds of serious injuries were significantly higher among males than females (AOR = 1.249, CI = 1.133–1.377), those in Grades 1–3 than 4–5 (AOR = 1.337, CI = 1.170–1.529), those who were mostly hungry (AOR = 1.299, CI = 1.024–1.648), current alcohol users (AOR = 1.283, CI = 1.136–1.449) and those who ever got drunk with alcohol (AOR = 1.200, CI = 1.055–1.365). Also, the risk of serious injuries was high among physically attacked victims (AOR = 1.756, CI = 1.568–1.965), those who engaged in physical fights (AOR = 1.642, CI = 1.468–1.838), those who were bullied outside school (AOR = 1.236, CI = 1.089–1.403), suicide attempters (AOR = 1.289, CI = 1.118–1.487), and those who had multiple sexual partners (AOR = 1.157, CI = 1.007–1.329).

**Conclusions:**

There is a high prevalence of serious injuries among in‐school adolescents in Bolivia. More prevention‐based policies and intervention programmes are required to decrease the occurrence of severe injuries among this cohort.

## 1 Introduction

Globally, around 1.2 billion people are adolescents aged 10–19 years, accounting for about one‐fourth of the global population [1]. In‐school adolescents participate in various activities, including risk‐taking behaviors like smoking, tobacco use, alcohol consumption, and risky sexual behaviors [2, 3, 4, 5]. These behaviors are responsible for most of the adverse health and social outcomes among this group [6, 7]. Among these risky behaviors, those resulting in serious injuries deserve particular attention due to their short‐ and long‐term effects on in‐school adolescents [8, 9, 10].

Serious injuries are a set of accidents which occur without forethought, leading to harmful effects for physical and psychological well‐being [11, 12]. Previous research reveals that injuries are a greater cause of premature morbidity and death among adolescents aged 10–19 years old [13, 14]. Injured adolescents may struggle with short‐ and long‐term physical, social, and mental health and may also experience reduced school performance [15, 16].

A growing body of evidence indicates that serious injuries among in‐school adolescents are both widespread and consistently high across diverse global settings, particularly in low‐ and middle‐income countries [17, 18, 19, 20]. Population‐based data suggest that these prevalences are not sporadic but represent a persistent public health concern, with estimates frequently exceeding one‐third of adolescents. For example, a multicountry study spanning 68 low‐ and middle‐income countries reported that approximately 42.9% of adolescents aged 12–15 years experienced at least one serious injury, indicating a substantial and common burden [19]. Similarly, high prevalence rates have been documented in Latin America, including Argentina, Uruguay, and Chile, suggesting regional consistency rather than isolated national trends [8]. Even more alarming levels have been reported in sub‐Saharan Africa, where an overall prevalence of 68.2% was observed across six countries, with country‐specific estimates remaining uniformly high (often exceeding 60% in Zambia, Kenya, Uganda, Zimbabwe, Namibia, and Swaziland [21]). These findings demonstrate that serious adolescent injuries occur at unacceptably high and relatively stable levels across regions, prompting the urgency of identifying context‐specific risk factors and informing targeted prevention strategies.

Serious injuries among in‐school adolescents can be attributed to many factors. These factors may include demographic (e.g., sex, age, grade), personal (e.g., truancy, hunger), drugs and substance use (e.g., use of amphetamine, marijuana use, alcohol use, ever got drunk), aggressiveness and violence (e.g., physically attacked, physical fight, and bullied), psychosocial (e.g., loneliness, multiple sexual partners, worry), suicidal behavior (e.g., ideation, plan, and attempt), and parental factors (e.g., understanding parents, parents knowledge on what adolescent does with free time) [8, 19, 22]. These factors may be explained from the socio‐ecological model (SEM) perspective. The SEM is based on the idea that an interaction exists between the individual and the environment, where the individual's behavior is determined to a large extent by the physical and social environment [23, 24].

Extant researchers using SEM found that (a) individual's behavior, (b) physical environment, and (c) social environmental factors could expose in‐school adolescents to serious injuries [25, 26]. Further evidence suggests that factors such as sociodemographic characteristics (being male and low socioeconomic status) [27], substance use (alcohol, tobacco, smoking, drugs) [28], psychological distress, depression, loneliness, being bullied, physical fights [19], truancy, ever had sex, parental or guardian support and bonding are associated with injuries among adolescents [29, 30, 31, 32].

Bolivia is a lower‐middle‐income, less urbanized country with a lower‐than‐average life expectancy, a higher infant and child mortality rate, and limited access to health care [33]. Consequently, risky behavior practices such as smoking cigarettes, consuming alcohol, physical fighting, carrying a weapon, attempting suicide, having sexual intercourse, and using illicit drugs among in‐school adolescents further increase their chances of adverse health outcomes, including injuries [22, 34, 35, 36]. However, information on these behavior patterns in Bolivia and their association with injuries is sparse.

Given the high rates of risky behaviors among the youth and adolescents in Bolivia, a better understanding of the serious injuries and risk factors is timely. Analysis of the factors influencing in‐school adolescent injuries using the SEM may help understand the complex web of injury occurrence and address specific issues by developing preventive interventions. Hence, this study examined the prevalence and associated factors of serious injuries among Bolivian in‐school adolescents. Understanding the distribution of injuries and associated factors among adolescents is crucial for educational campaigns, evidence‐based planning, priority setting and resource allocation for prevention and control in Bolivia.

## 2 Materials and Methods

### 2.1 Study Design and Setting

The 2018 Bolivia Global School‐Based Student Health Survey (GSHS) is a cross‐sectional study conducted among school‐going adolescents in the Plurinational State of Bolivia. This survey used a self‐administered questionnaire to collect data on adolescents' health behaviors and protective factors in Bolivia. Data collected from participants in Bolivia's various secondary school settings can be accessed via the link “https://extranet.who.int/ncdsmicrodata/index.php/catalog/881.” The GSHS in Bolivia involved a school‐based survey conducted among students in 2nd secondary to 6th secondary, which are typically attended by students aged 13–17 [37].

### 2.2 Ethical Consideration

The Bolivian Ministry of Health secured ethical clearance as the study's primary investigator. Using the survey identity: “BOL_2018_GSHS_v01,” the Ministry of Health collaborated with the World Health Organization (WHO) and the Centers for Disease Control and Prevention (CDC) to conduct the study. Entry permission was obtained from the Bolivian Ministry of Education before the recruitment of the schools. Permission was also obtained from the heads and teachers of the various schools prior to data collection. Given that the study participants were aged 13–17, parental approval and child assent were obtained. All ethical requirements were adhered to throughout data collection, as well as the Helsinki Declaration for conducting studies involving human subjects.

### 2.3 Sampling

A weighted sample of 7822 students in secondary schools in Bolivia who were in Grades 2 through 6 was used in this study. These samples consisted of students aged 13–17. Data were obtained using a two‐stage cluster sampling technique to make the data representative of all Bolivian students in secondary Grades 2 through 6. Initially, schools were chosen with a probability inversely correlated with students' enrollment. Classes were randomly selected for the second round, and every student in those classes was recruited to participate in the study. The response rates for the various geographical coverages are as follows, “National: School response (94%), student response rate (84%), and overall response rate (79%); Highland: School response rate (100%), student response rate (88%), and overall response rate (88%); Plains: School response rate (97%), student response rate (83%), and overall response rate (80%); Valley: School response rate (84%), student response rate (81%), and overall response rate (68%).”

### 2.4 Study Variables

The study's predicted variable is “self‐reported serious injuries,” defined as “whether or not the student was seriously injured one or more times during 12 months before the survey.” An injury was defined as serious in this study when it resulted in a student missing at least one full day of usual activities (such as school, sports, or a job) or requiring treatment by a doctor or nurse [37]. A score of “1” was given to every response of “Yes” while a score of “0” was given to “No” responses. The demographic variables (sex, age, grade), personal factors (truancy, hunger), drugs and substance use (amphetamine use, marijuana use, current alcohol use, ever got drunk), aggressive and violent behaviors (physically attacked, physical fight, bullied on school property, bullied outside the school property, cyberbullied), psychosocial factors (loneliness, multiple sexual partners, worry), suicidal behavior (suicide ideation, suicide plan, suicide attempt), and parental factors (understanding parents, parental knowledge of what adolescent does with free time) constituted the predictor variables. Each predictor variable was coded such that a response of “Yes” was scored 1, while “No” responses were scored “0.”

### 2.5 Data Analysis

The SPPS version of the GSHS microdata was obtained from the WHO website “https://extranet.who.int/ncdsmicrodata/index.php/catalog/881” and cleaned for analysis using the IBM SPSS software version 21. We applied sample weighting at the school, student, and sex within grade levels to ensure representative results and minimize bias due to patterns and nonresponses. Also, to address missing values in the data, we used the multiple imputation technique. This technique was applied to variables with missing values exceeding 1% but < 10% and missing at random [12]. Five imputations were produced using the automatic imputation method to maintain data quality [38]. We compared the imputed values with the observed values using a complete‐case analysis. A bivariate analysis using Pearson's *χ* ^2^ was used to examine the relationship between severe injuries and the independent variables (see Table 1). Significant variables (*p* < 0.05, two‐sided at 95% confidence interval (CI)) at the bivariate level were included in a binomial logistic regression model (see Table 2). The Hosmer–Lemeshow test was conducted to assess the goodness‐of‐fit of the regression model, and the results revealed no evidence of lack of fit with our model's attempt to predict serious injuries. Regression results were presented using 95% CIs and adjusted odds ratios (AORs) with a significance level of *p* < 0.05.

**Table 1:** Bivariate analysis examining the association between serious injuries and independent variables (*n* = 7822).

Variables	Response	Serious injuries Injuries (%)	No injuries (%)	Chi‐square (χ²)	*φ*
**Demographic**					
Sex	Male	2243 (55.7%)	1785 (44.3%)	54.85***	−0.084
	Female	1795 (47.3%)	1999 (52.7%)		
Age	13–15	2020 (51.7%)	1884 (48.3%)	.044	−0.002
	16–17	2018 (51.5%)	1900 (48.5%)		
Grade	Grade 1–3	2687 (53.3%)	2353 (46.7%)	16.20***	0.046
	Grades 4–5	1351 (48.6%)	1431 (51.4%)		
**Personal**					
Truancy	Yes	1802 (56.1%)	1412 (43.9%)	43.14***	−0.074
	No	2236 (48.5%)	2372 (51.5%)		
Hunger	Yes	218 (63.7%)	124 (36.3%)	21.03***	−0.051
	No	3820 (51.1%)	3660 (48.9%)		
**Drugs and substance use**					
Use of amphetamine	Yes	381 (64.3%)	212 (35.7%)	40.96***	−0.076
	No	3657 (50.6%)	3572 (49.4%)		
Marijuana use	Yes	497 (65.4%)	263 (34.6%)	63.92***	−0.076
	No	3541 (50.1%)	3521 (49.9%)		
Current alcohol use	Yes	1352 (62.2%)	821 (37.8%)	135.23***	−0.131
	No	2686 (47.5%)	2963 (52.5%)		
Ever got drunk	Yes	1242 (62.0%)	760 (38.0%)	116.85***	−0.123
	No	2796 (48.0%)	3024 (52.0%)		
**Aggression and violence**					
Physically attacked	Yes	1498 (67.2%)	731 (32.8%)	303.05***	−0.197
	No	2540 (45.4%)	3053 (54.6%)		
Physical fight	Yes	1591 (66.8%)	791 (33.2%)	315.57***	−0.201
	No	2447 (45.0%)	2993 (55.0%)		
Bullied on school property	Yes	1205 (59.2%)	832 (40.8%)	62.58***	−0.089
	No	2833 (49.0%)	2952 (51.0%)		
Bullied outside school property	Yes	1104 (62.6%)	660 (37.4%)	109.58***	−0.118
	No	2934 (48.4%)	3124 (51.6%)		
Cyberbullied	Yes	1059 (60.0%)	705 (40.0%)	64.51***	−0.091
	No	2979 (49.2%)	3079 (50.8%)		
**Psychosocial**					
Loneliness	Yes	872 (56.7%)	667 (43.3%)	19.46***	0.050
	No	3166 (50.4%)	3117 (49.6%)		
Multiple sexual partners	Yes	795 (61.8%)	491 (38.2%)	64.07	0.091
	No	3243 (49.6%)	3293 (50.4%)		
Worry	Yes	675 (58.5%)	479 (41.5%)	25.57	0.057
	No	3363 (50.4%)	3305 (49.6%)		
**Suicidal behaviour**					
Suicidal ideation	Yes	1078 (58.7%)	760 (41.3%)	47.51***	0.078
	No	2960 (49.5%)	3024 (50.5%)		
Suicide plan	Yes	1004 (60.0%)	670 (40.0%)	59.50***	.087
	No	3034 (49.3%)	3114 (50.7%)		
Suicide attempt	Yes	1093 (63.1%)	638 (36.9%)	118.108***	0.123
	No	2945 (48.4%)	3146 (51.6%)		
**Parental factors**					
Understanding parents	Yes	1195 (49.0%)	1246 (51.0%)	10.12**	−0.036
	No	2843 (52.8%)	2538 (47.2%)		
Parents’ knowledge of what adolescents do with their free time	Yes	1440 (48.2%)	1550 (51.8%)	23.24***	−0.055
	No	2598 (53.8%)	2234 (46.2%)		

*Note:* Percentages represent row percentages. χ² = Chi‐square test statistic; *φ* = Phi coefficient (effect size).

** *p* < .01

*** *p* < .001.

**Table 2:** Association between variables that were significant and severe injuries in adolescents.

Variables	*B*	Wald test (*z*‐ratio)	Odds ratio	95% confidence interval for odds ratio	
				Lower	Upper
Demographic					
Sex (male)	0.223	20.035	1.249***	1.133	1.377
Age (13–15)	−0.109	2.752	0.897	0.789	1.020
Grade (1–3)	0.291	18.078	1.337***	1.170	1.529
Personal					
Truancy	0.087	3.062	1.091	0.990	1.203
Hunger	0.262	4.646	1.299*	1.024	1.648
Drugs and substance use					
Amphetamines or methamphetamines use	0.034	0.108	1.034	0.845	1.267
Current marijuana use	−0.039	0.167	0.962	0.797	1.161
Current alcohol use	0.249	16.104	1.283***	1.136	1.449
Ever got drunk	0.182	7.741	1.200**	1.055	1.365
Aggression and violence					
Physical attack	0.563	95.720	1.756***	1.568	1.965
Physical fight	0.496	75.000	1.642***	1.468	1.838
Bullied on school property	0.081	1.828	1.084	0.964	1.220
Bullied outside school property	0.212	10.734	1.236**	1.089	1.403
Cyberbullied	0.120	3.760	1.127	0.999	1.272
Psychosocial					
Loneliness	−0.006	0.007	0.994	0.874	1.132
Multiple sexual partners	0.146	4.257	1.157*	1.007	1.329
Worry	0.058	0.632	1.060	0.918	1.224
Suicidal behavior					
Suicide ideation	−0.033	0.204	0.968	0.839	1.116
Suicide plan	0.035	0.216	1.035	0.895	1.197
Attempted suicide	0.254	12.169	1.289***	1.118	1.487
Parental					
Understanding parents	0.005	0.009	1.005	0.902	1.121
Parental knowledge of what adolescent does with their free time	−0.014	0.066	0.986	0.889	1.095

*Note:* Hosmer and Lemeshow test was conducted to assess the goodness of fit, and the result showed *χ* ^2^ (8) = 3.536, with a *p* value of 0.896. The asterisks (*) indicate statistical significance levels, where

* *p* < 0.05;

** *p* < 0.01;

*** *p* < 0.001.

## 3 Results

### 3.1 Background Characteristics of Participants

**Figure 1:**
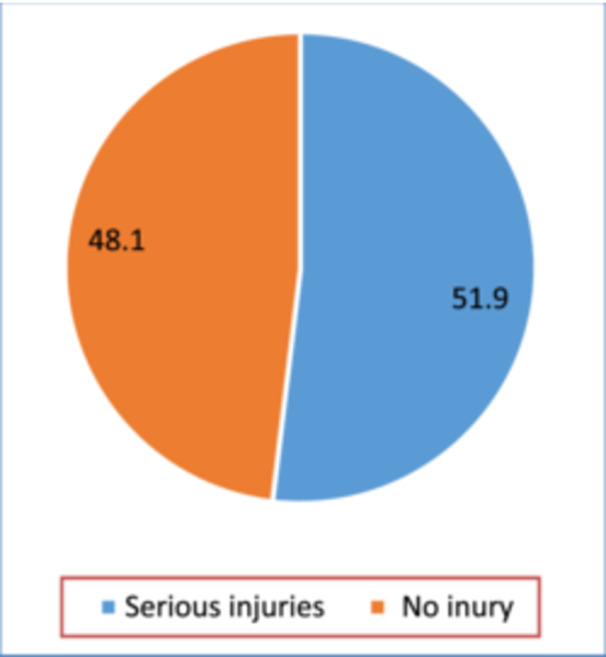
Occurrence of severe injuries among adolescents in Bolivia.

Figure 1 shows that the prevalence of serious injuries among Bolivian in‐school adolescents was 51.9%. The majority of those who experienced serious injuries were males (55.5%). Also, half (50%) of the participants aged 13–15 and 16–17 experienced serious injuries. Moreover, 66.5% of those who experienced serious injuries were in Grades 1–3. Furthermore, 44.7% of serious injury victims were truants. Also, 9.4% of amphetamine, 12.3% of marijuana, and 33.5% of alcohol users experienced serious injuries. Again, 37.1% of physically attacked victims, 39.4% of those who engaged in physical fights, and 29.8% of those bullied on school property experienced serious injuries. Further, 21.6% of lonely victims, 26.7% of those who had suicidal ideation, 24.9% of those who planned suicide, and 27.1% of suicide attempters experienced serious injuries. Further, 29.6% and 35.7% of participants whose parents understood them and those whose parents knew what they did with their free time experienced serious injuries, respectively (see Table 1).

### 3.2 Distribution and Chi‐Square Analysis of Serious Injuries

Table 1 presents the bivariate analysis of the study. The results show that demographic characteristics, such as sex (*χ* ^2^ = 54.85, *p*< 0.001) and grade (*χ* ^2^ = 16.20, *p*< 0.001), were significantly associated with serious injuries. Personal characteristics of participants, such as school truancy (*χ* ^2^ = 43.14, *p* < 0.001) and hunger (*χ* ^2^ = 21.03, *p* < 0.001), were significantly associated with serious injuries. Amphetamine use (*χ* ^2^ = 40.96, *p* < 0.001), marijuana use (*χ* ^2^ = 63.92, *p* < 0.001), alcohol use (*χ* ^2^ = 135.23, *p* < 0.001), and ever getting drunk with alcohol (*χ* ^2^ = 116.85, *p* < 0.001) were significantly associated with serious injuries. Furthermore, being physically attacked (*χ* ^2^ = 303.05, *p* < 0.001), engaging in physical fights (*χ* ^2^ = 315.57, *p *< 0.001), being bullied on (*χ* ^2^ = 62.58, *p* < 0.001) and off (*χ* ^2^ = 109.58, *p* < 0.001) school property, and being cyberbullied (*χ* ^2^ = 64.51, *p* < 0.001) were significantly associated with serious injuries. Other factors such as loneliness, worry, suicidal behaviors (ideation, plan, and attempt), understanding parents, and parental knowledge of what adolescents do with free time were significantly associated with serious injuries (see Table 1).

### 3.3 Logistic Regression Examining the Correlates of Serious Injuries Among In‐School Adolescents

Table 2 presents the binomial logistic regression analysis on the determinants of serious injuries among participants. The odds of serious injuries was significantly high among males than females (AOR = 1.249, CI = 1.133–1.377), those in Grade 1–3 than 4–5 (AOR = 1.337, CI = 1.170–1.529), those who were mostly hungry (AOR = 1.299, CI = 1.024–1.648), current alcohol users (AOR = 1.283, CI = 1.136–1.449) and those who got drunk with alcohol (AOR = 1.200, CI = 1.055–1.365). Again, the risk of serious injuries was high among physically attacked victims (AOR = 1.756, CI = 1.568–1.965), those who engaged in physical fights (AOR = 1.642, CI = 1.468–1.838), those who were bullied outside school (AOR = 1.236, CI = 1.089–1.403), suicide attempters (AOR = 1.289, CI = 1.118–1.487), and those who had multiple sexual partners (AOR = 1.157, CI = 1.007–1.329) (see Table 2).

## 4 Discussion

This study found that more than half (51.9%) of in‐school adolescents in Bolivia had sustained a serious injury within the past year, highlighting a widespread and persistent global public health challenge rather than a context‐specific anomaly. This finding aligns with previous studies conducted in diverse global settings [18, 19, 20], reaffirming that adolescent injuries remain a persistent issue in both high‐ and low‐income contexts. The high injury prevalence in Bolivia reflects broader regional trends in South and Central America, where similar sociodemographic and economic profiles, such as in Argentina, Uruguay, and Chile, have been linked with elevated rates of adolescent violence and injury [8]. Likewise, parallels with findings from sub‐Saharan Africa suggest that shared health‐risk behaviors, limited preventive infrastructures, and constrained adolescent‐focused intervention tools in many low‐ and middle‐income countries contribute to remarkably similar injury burdens across settings [21, 31, 39]. Injuries among adolescents can contribute to long‐term disability, reduced academic attainment, psychological distress, and increased healthcare utilization. If preventive measures are not implemented, Bolivia's educational and health systems may face growing strain from youth morbidity.

The present study found that male adolescents were significantly more likely to experience serious injuries. The higher injury rates among male adolescents align with previous evidence indicating that boys are more likely to engage in physical aggression, risk‐taking, and unsafe activities [40, 41]. This finding may be influenced by sociocultural norms that promote toughness or dominance in males. Over time, unaddressed gender norms and behavioral risks may entrench cycles of violence and health inequalities, especially if schools and communities fail to provide gender‐sensitive interventions that promote emotional intelligence and conflict resolution among male students.

Younger students in lower grades (Grades 1–3) were more likely to sustain injuries than their senior counterparts, possibly due to lower emotional regulation, underdeveloped risk perception, or inadequate supervision. Repeated injuries at a young age can disrupt early educational progress and undermine foundational learning. This finding underscores the need for early‐age interventions, including supervised recreation, life skills training, and age‐appropriate safety education, to minimize the long‐term effects of early‐life injury.

Several health‐risk behaviors were identified as strong predictors of injury, including hunger, alcohol use, and experiencing drunkenness. The association between food insecurity and injury may reflect broader household vulnerabilities and social instability that expose adolescents to violence [11]. Hunger may also impair concentration and coping skills, potentially increasing conflict and risk behaviors. Alcohol use, on the other hand, has been widely documented to impair judgment and increase aggression, thereby contributing to both unintentional and intentional injuries [42, 43]. These findings reinforce the importance of cross‐sectoral policies addressing adolescent nutrition, substance abuse, and economic inequalities.

The study further found that aggressive and violent behaviors such as being physically attacked, participating in fights, and experiencing bullying outside of school were strongly associated with injury. These behaviors often form part of broader patterns of adolescent violence and social exclusion, which not only increase physical harm but also contribute to poor mental health and academic disengagement [27, 28]. Also, adolescents in violent school environments are at risk for chronic trauma and disengagement from education. These experiences may normalize aggressive behaviors, perpetuating a cycle of harm into adulthood.

Injuries were also prevalent among Bolivian in‐school adolescents who had multiple sexual partners. Like other health‐risk behaviors, risky sexual behaviors have been linked with injuries in many studies [44, 45, 46]. Generally, injuries related to intimate partner and sexual violence are more likely to increase among adolescents who often engage in multiple sexual partner relationships. Another vital risk factor for injuries in the current work was suicide attempts among Bolivian in‐school adolescents. This finding was not surprising because Dearden et al. [34] earlier found that suicide attempts were high among Bolivian adolescents. According to Dearden et al. [34], a high prevalence of suicide attempts correlates with being threatened or injured with weapons. Thus, adolescents who attempt suicide are most likely to harm themselves, leading to severe injuries. Other recent studies have also found significant associations between suicidal behaviors and injuries among adolescents [12, 47].

Situating the study's results within the context of SEM can well explain the causes of unintended injuries among in‐school adolescents in Bolivia. In line with SEM research, injuries among in‐school adolescents are associated with multiple factors that may be complexly linked to the individual, physical, and social environments [25, 26]. Knowing the associated factors, as indicated in several previous studies, is the first step toward reducing adolescent injuries in Bolivia [21, 29, 31, 32, 48, 49, 50].

### 4.1 Strengths and Limitations

The timely and comprehensive analysis of serious injury using a large sample size and standardized procedures for the selection of participants offers a generalization of findings to in‐school adolescents in Bolivia. Besides, direct comparisons of variables and prevalence estimates provide current trends on the phenomenon for planned interventions. Despite these strengths, the study has some limitations. It is possible that some adolescents with injuries were not captured in the GSHS because it only includes pupils enrolled in school at the time of the survey. It is also challenging to determine the causal relationship between injuries and different risk behaviors and other factors, as the survey is self‐reported and cross‐sectional, which excludes students' injury reports from verification by parents or medical records. Self‐reporting also introduces the potential for recall bias and misinterpretation of survey questions. In addition, the use of binary (yes/no) response options for key risk behaviors may oversimplify complex behavioral patterns, potentially leading to more extreme interpretations of associations. For example, adolescents who experimented with substances such as amphetamines on a single occasion are classified in the same category as chronic users, despite likely differences in exposure intensity and risk. This lack of information on frequency, duration, or severity may have resulted in residual misclassification and overestimation of associations. Furthermore, other sources of bias, including variation in data quality, incomplete responses, and possible underreporting across geographic areas, cannot be ruled out entirely. Nonetheless, the GSHS provides valuable population‐level insights into injury‐related risk factors and remains a helpful resource for informing adolescent injury prevention and health promotion strategies.

### 4.2 Implications

The study's findings suggest a high prevalence of severe injuries among young people in Bolivia. This implies that more prevention‐based policies and programmes should be urgently developed to decrease severe injuries among in‐school adolescents in Bolivia. This study provides further evidence of factors associated with severe injuries among in‐school adolescents in Bolivia. Having identified these risk factors, the study paves the way for the effective design of intervention programmes to address severe injury challenges among Bolivian adolescents through health education and targeted interventions. Developing interventions to address these identified factors would prevent severe injuries that can lead to morbidity and mortality among the Bolivian adolescent population.

## 5 Conclusions

In‐school adolescents in Bolivia between the ages of 13 and 15 continue to have a high rate of serious injuries, particularly among boys. Further, the causes of these injuries included multiple factors such as demographic, personal, drug and substance usage, aggressiveness and violence, psychological, suicidal behavior, and lack of parental supervision. Using SEM as a framework, a timely, thorough, and in‐depth analysis of injuries among Bolivian adolescents may help academics, policymakers, and other decision‐makers identify the myriad associated factors. More so, unmet needs and multidisciplinary efforts may help stakeholders implement essential school health promotion programmes to prevent serious injuries among this cohort.

## Author Contributions

Conceptualization: Jacob Owusu Sarfo. Methodology, data curation, data analysis: Jacob Owusu Sarfo, Paul Obeng, Newton Isaac Gbordzoe, Richmond Stephen Sorkpor, Edmond Kwesi Agormedah, Medina Srem‐Sai, and John Elvis Hagan. Writing – original draft preparation. writing – review and editing: Jacob Owusu Sarfo and John Elvis Hagan. Writing – supervision: Jacob Owusu Sarfo. Funding: John Elvis Hagan. All authors have read and approved the final version of the manuscript (the corresponding author had full access to all of the data in this study and takes complete responsibility for the integrity of the data and the accuracy of the data analysis).

## Funding

The authors received no specific funding for this work.

## Disclosure

The lead author Jacob Owusu Sarfo affirms that this manuscript is an honest, accurate, and transparent account of the study being reported; that no important aspects of the study have been omitted; and that any discrepancies from the study as planned (and, if relevant, registered) have been explained.

## Ethics Statement

The authors declare that all methods used in this project complied with the Helsinki Declaration. The Institutional Review Boards of the WHO, CDC, and the Ministry of Health and Education of Bolivia approved the study's ethical standards, with a survey identifier: “BOL_2018_GSHS_v01.”

## Consent

All individuals who participated in the study provided written consent.

## Conflicts of Interest

The authors declare no conflicts of interest.

## Data Availability

The authors used data from the GSHS in this study, which is freely available and funded by the US CDC and WHO. The WHO repository has this data set available for free download at “https://extranet.who.int/ncdsmicrodata/index.php/catalog/881.”
